# A histopathological overlap of linear IgA bullous dermatosis and vasculitis

**DOI:** 10.1016/j.jdcr.2024.07.037

**Published:** 2024-08-24

**Authors:** Tharani Murali, Le Wen Chiu, Nicole L. Bolick, Emily Altman, Julie Harrington, Charles M. Phillips

**Affiliations:** a University of New Mexico School of Medicine, Albuquerque, New Mexico; b Department of Dermatology, University of New Mexico School of Medicine, Albuquerque, New Mexico; c Department of Pathology & Laboratory Medicine Service, Raymond G. Murphy Department of Veterans Affairs Medical Center, Albuquerque, New Mexico; d Department of Dermatology, Raymond G. Murphy Department of Veterans Affairs Medical Center, Albuquerque, New Mexico

**Keywords:** linear IgA bullous dermatosis, vancomycin, vasculitis

## Introduction

Linear IgA bullous dermatosis (LABD) is a rare autoimmune vesiculobullous disease characterized by the linear deposition of IgA along the cutaneous basement membrane zone. LABD has been shown to be associated with various autoimmune diseases, malignancies, gastrointestinal diseases, and infections.^1^, ^2^, ^3^ There are several cases of drug-induced LABD in the literature with vancomycin being the most common offending agent.^4^

Leukocytoclastic vasculitis (LCV) is a small vessel vasculitis characterized by deposition of immune complexes in vessel walls. It is most often idiopathic in nature but can be induced by infections or certain medications such as beta-lactam antibiotics, sulfonamides, phenytoin, and allopurinol.^5^ There are very few cases reported in the literature of a presentation of both LABD and LCV.^6^^,^^7^ Here, we present a case of a vancomycin-induced bullous eruption with histopathology showing features of the sublamina densa subtype of LABD and LCV.

## Case report

**Fig 1:**
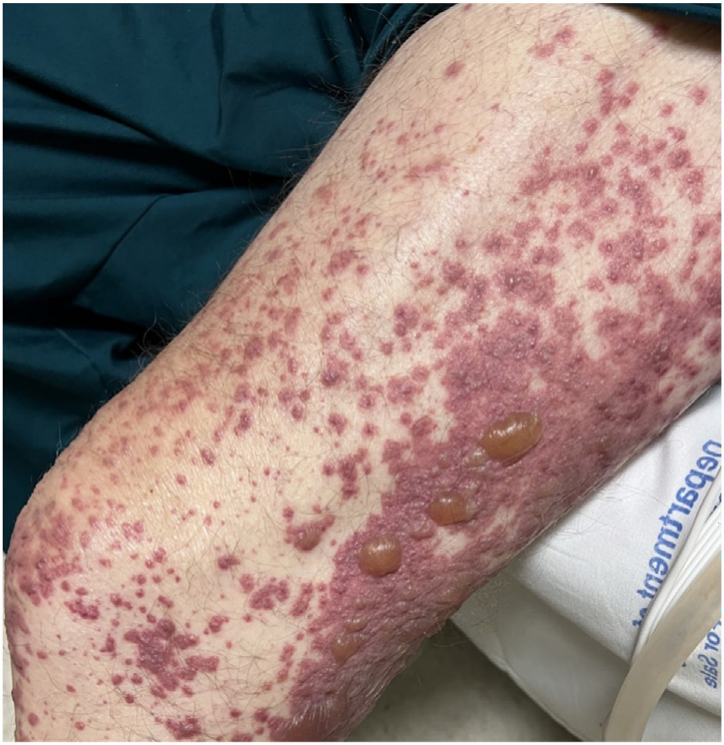
Palpable purpura with bullae on left lower extremity.

**Fig 2:**
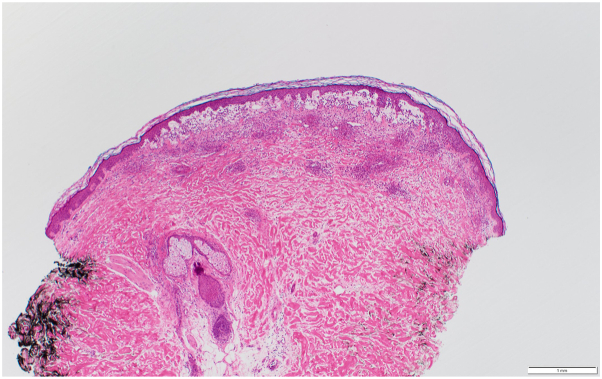
Low-power view of fibrinoid necrosis, stained with H&E. Magnification: ×40.

**Fig 3:**
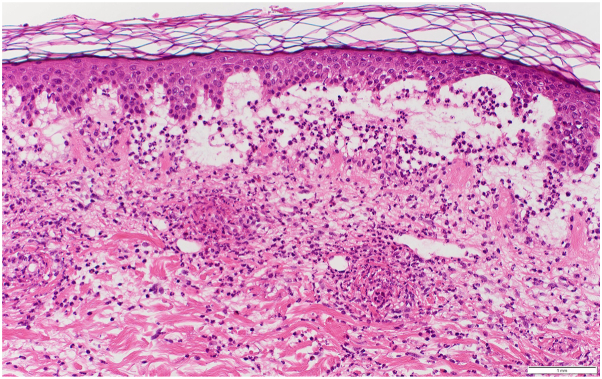
H&E stain of a subepidermal vesicle with neutrophils and fibrinoid necrosis of vessel walls with fragments of neutrophils. Magnification: ×200.

**Fig 4:**
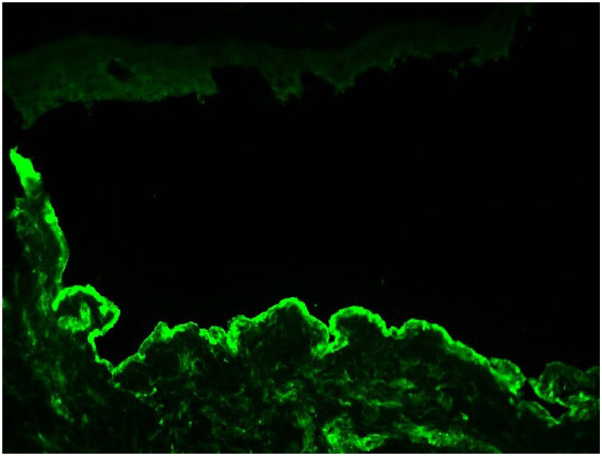
Direct immunofluorescence of thick linear IgA deposition along the cutaneous basement membrane zone.

A 74-year-old male with a history of squamous cell carcinoma of the lung was admitted to the hospital for a pneumothorax and empyema, status post-right posterolateral thoracotomy and right upper lobectomy. He was taking piperacillin/tazobactam and vancomycin for 1 week when dermatology was consulted for a rash. On examination, the patient had deeply purpuric macules to papules that were coalescing with scattered bullae on the bilateral lower extremities, lower back, and abdomen (Fig 1). Punch biopsies were performed on the right abdomen, and histopathology showed a subepidermal vesicle filled with neutrophils, consistent with LABD (Figs 2 and 3). However, it also showed a superficial perivascular neutrophilic infiltrate with occasional eosinophils in addition to fibrinoid necrosis of superficial vessels (Fig 3). There was no red blood cell extravasation or leukocytoclasia. The direct immunofluorescence (DIF) showed a thick linear IgA deposition along the epidermal basement membrane zone (Fig 4). The thick linear staining of IgA was localized at the dermal floor of the salt split skin, indicative of the sublamina densa subtype of LABD. There was an absence of IgG, IgM, C3, C5b-9, or fibrinogen deposition. Vancomycin was discontinued and the patient was started on a 60-milligram taper of prednisone with reduction of 10 milligrams every 3 days in addition to topical triamcinolone. The patient returned for a follow-up appointment 2 months later with resolution of the rash.

## Discussion

LABD and LCV are separate disease entities with very few cases of overlap reported in the literature.^6^^,^^7^ Clinically, they have distinct presentations. LABD classically presents with subepidermal tense bullae that appear in a herpetiform-like arrangement. LCV most commonly presents as palpable purpura on the lower extremities, which is more consistent with the presentation seen in the present patient.

The pathophysiology of the 2 conditions also differs. LABD can be divided into 2 different subtypes: lamina lucida and sublamina densa. In the sublamina densa subtype, which is rare, IgA antibodies bind to type VII collagen within anchoring fibrils, specifically COL7.^8^ In LCV, immune complexes are deposited in vessel walls leading to complement activation. Both conditions are commonly idiopathic in nature but can have various etiologies including drug exposure.^4^^,^^5^ Vancomycin is a common offending agent for LABD, but it is rarely associated with LCV.^4^^,^^9^ Given that the eruption of the rash occurred shortly after initiating vancomycin treatment and that the rash resolved upon discontinuation of vancomycin, it was deduced that vancomycin was the cause of this patient’s rash. It is possible that this patient developed 2 concurrent rashes from piperacillin/tazobactam and vancomycin; however, this is very unlikely given the limited literature on the concurrence of 2 different drug rashes.

Histologically, LABD is characterized by a subepidermal blister filled with neutrophils and linear IgA deposition along the cutaneous epidermal basement membrane zone on DIF. LCV is characterized by neutrophilic infiltration of vessel walls and fibrinoid necrosis with DIF showing deposition of C3, IgM, IgG, and/or IgA in a granular pattern.

Our patient’s pathology showed features of both LABD and LCV. The histopathology showed fibrinoid necrosis of vessel walls consistent with LCV and a subepidermal vesicle with neutrophils consistent with LABD. However, the DIF only showed linear IgA deposition at the basement membrane and no granular immunoglobulin deposition within the vessel walls. In summary, the clinical presentation of this patient is most consistent with LCV, but the pathology suggests an overlap of LABD and LCV. A prior case reported in the literature showed histopathology consistent with LCV and DIF consistent with both LABD and LCV.^7^ Another case had classic features of LABD both clinically and histopathologically but had an additional vasculitic component histologically.^7^ The etiology of this overlap is unknown. It has been speculated that there is a new IgA antibody that binds to both the epidermal and vascular basement membrane zones causing this overlap.^6^ Alternatively, “epitope spreading” could explain the IgA antibody binding to both epidermal and vascular basement membrane zones.^10^ Another study hypothesized that the circulating IgA antibody present in LABD could activate the complement cascade, leading to vasculitis.^7^ In our patient, it is unclear if this is a primary case of vasculitis or if it is LABD with vasculitic changes. While it is possible this could be a case of concomitant LABD and LCV induced by 2 different medications, there are limited cases of LABD and LCV in the literature, let alone concomitant LABD and LCV caused by 2 separate medications. Nonetheless, our case provides supporting evidence for a hybrid or overlap entity termed “linear vasculitis” and is interesting as this patient had the sublamina densa subtype of LABD, which may contribute to this unique presentation of LABD and LCV.^7^

## Conflicts of interest

None disclosed.
